# Supplementary material for: Single cell multi-omics reveal intra-cell-line heterogeneity across human cancer cell lines
Source: Nat Commun. 2023 Dec 9;14:8170. doi: 10.1038/s41467-023-43991-9 (PMC10710513; doi:10.1038/s41467-023-43991-9)
Supplement: Supplementary file 1 — Supplementary Information [file 41467_2023_43991_MOESM1_ESM.pdf]

Supplementary information to:

Single cell multi-omics reveal intra-cell-line heterogeneity across human cancer cell lines

Qionghua Zhu[1,2,14,*], Xin Zhao[3,4,14], Yuanhang Zhang[3,4,14], Yanping Li[2,14], Shang Liu[3,14], Jingxuan Han[2], Zhiyuan Sun[2], Chunqing Wang[3,4], Daqi Deng[2], Shanshan Wang[3], Yisen Tang[2], Yaling Huang[3], Siyuan Jiang[3,4], Chi Tian[2], Xi Chen[3], Yue Yuan[3], Zeyu Li[3,4], Tao Yang[5], Tingting Lai[5], Yiqun Liu[5], Wenzhen Yang[5], Xuanxuan Zou[3,4], Mingyuan Zhang[3], Huanhuan Cui[1,2,6], Chuanyu Liu[3], Xin Jin[3], Yuhui Hu[1,2,7], Ao Chen[3,8,12], Xun Xu[3], Guipeng Li[1,2,6], Yong Hou[3,9], Longqi Liu[3,10,11,*], Shiping Liu[3,9,10,11,12,*], Liang Fang[1,2,6,*], Wei Chen[1,2,*], Liang Wu[3,8,13,*]

1. Shenzhen Key Laboratory of Gene Regulation and Systems Biology, School of Life Sciences, Southern University of Science and Technology, Shenzhen 518055, China

2. Department of Systems Biology, School of Life Sciences, Southern University of Science and Technology, Shenzhen 518055, China

3. BGI Research, Shenzhen 518083, China

4. College of Life Sciences, University of Chinese Academy of Sciences, Beijing 100049, China

5. China National GeneBank, Shenzhen 518120, China

6. Academy for Advanced Interdisciplinary Studies, Southern University of Science and Technology, Shenzhen 518055, China

7. Department of Pharmacology, School of Medicine, Southern University of Science and Technology, Shenzhen 518055, China

8. JFL-BGI STOmics Center, Jinfeng Laboratory, Chongqing 401329, China

9. Shenzhen Key Laboratory of Single-Cell Omics, BGI-Shenzhen, Shenzhen 518100, China

10. BGI Research, Hangzhou 310012, China

11. Shenzhen Bay Laboratory, Shenzhen 518000, China

12. The Guangdong-Hong Kong Joint Laboratory on Immunological and Genetic Kidney Diseases

13. BGI Research, Chongqing 401329, China

14. These authors contributed equally: Qionghua Zhu, Xin Zhao, Yuanhang Zhang, Yanping Li, Shang Liu

*Correspondence: zhuqh@mail.sustech.edu.cn (Q.Z.) liulongqi@genomics.cn (L.L.) liushiping@genomics.cn (S.L.) fangl@sustech.edu.cn (L.F.) chenw@sustech.edu.cn (W.C.) wuliang@genomics.cn (L.W.)

Supplementary Fig. 1

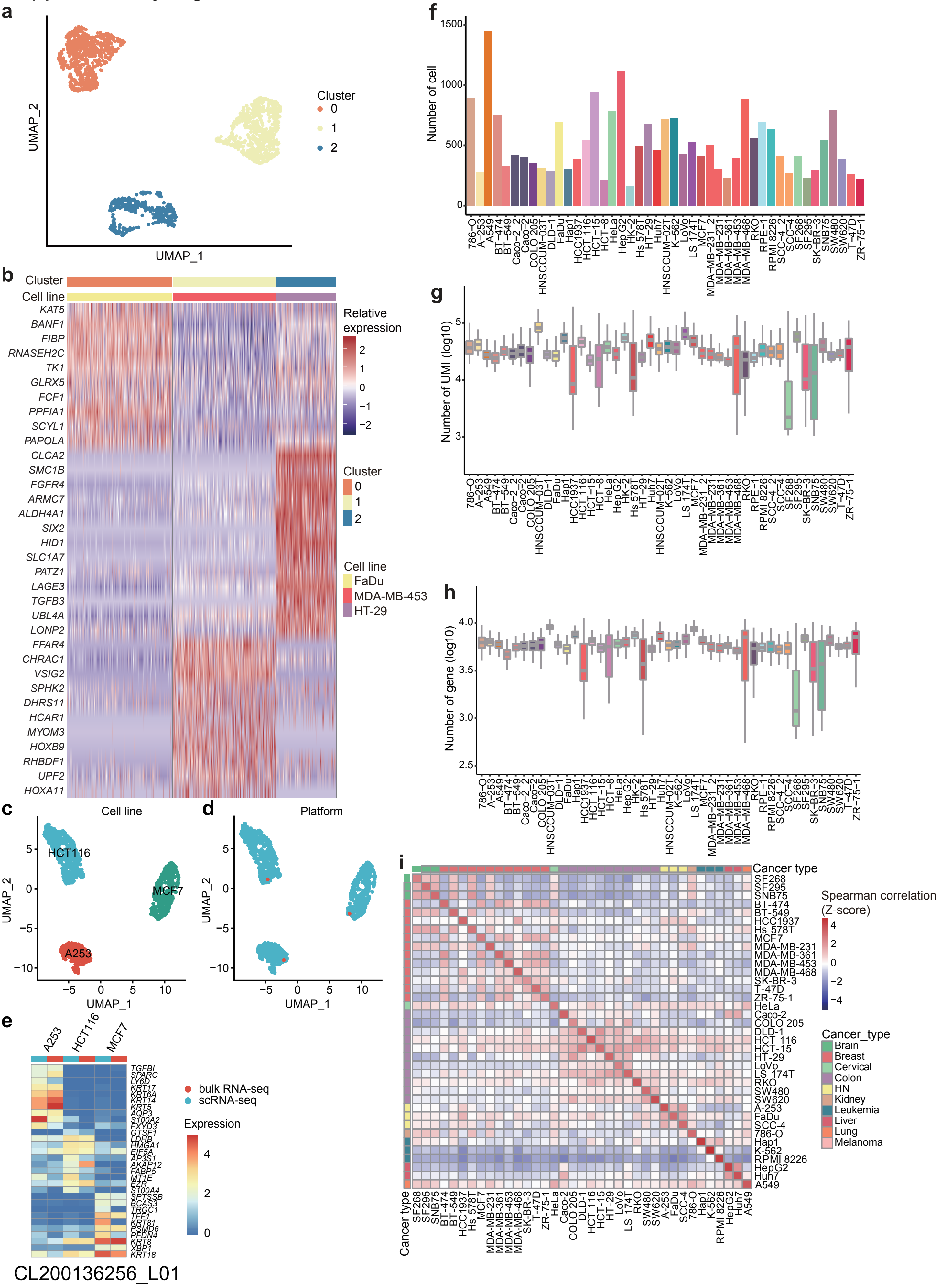

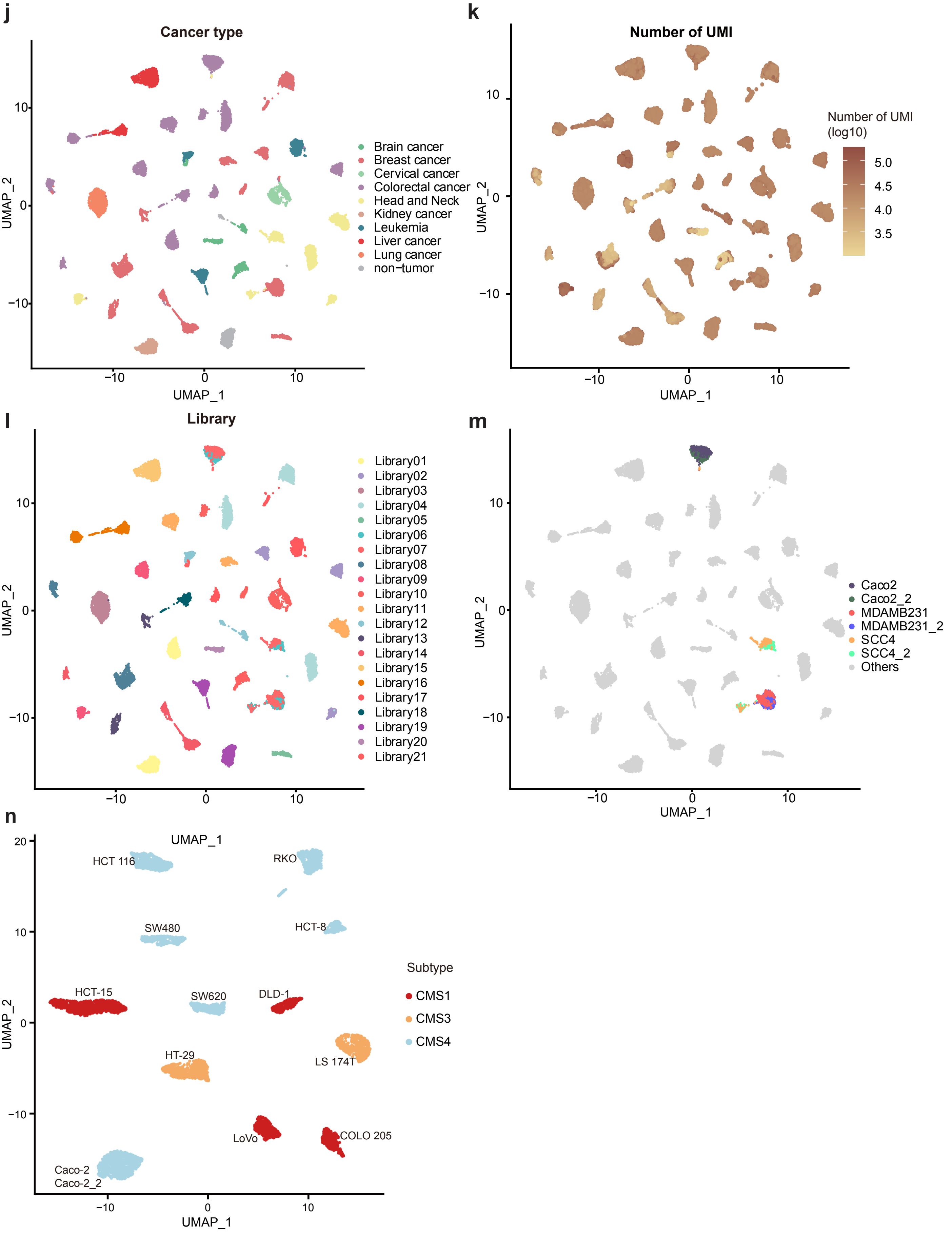
j
Cancer type
UMAP_2
UMAP_1
Brain cancer
Breast cancer
Cervical cancer
Colorectal cancer
Head and Neck
Kidney cancer
Leukemia
Liver cancer
Lung cancer
non−tumor
k
Number of UMI
Number of UMI (log10)
5.0
4.5
4.0
3.5
l
Library
Library01
Library02
Library03
Library04
Library05
Library06
Library07
Library08
Library09
Library10
Library11
Library12
Library13
Library14
Library15
Library16
Library17
Library18
Library19
Library20
Library21
m
Caco2
Caco2_2
MDAMB231
MDAMB231_2
SCC4
SCC4_2
Others
n
HCT 116
RKO
SW480
HCT-8
HCT-15
SW620
DLD-1
HT-29
LS 174T
LoVo
COLO 205
Caco-2
Caco-2_2
Subtype
CMS1
CMS3
CMS4

Supplementary Figure 1. Quality control of the scRNA-seq data set.

a, UMAP plot of a representative mix demonstrating the cell line assignment. **b,** Cells were clustered by their global expression profiles. **c,** UMAP plot showing the three cell lines in the batch of CL200136256_L01, labeled by different colors. **d,** UMAP plot showing the resource of each spot, with cells in blue from scRNA-seq and red from bulk RNA-seq. **e,** Heatmap depicting the expression of the top 10 marker genes of the three cell lines based both on scRNA-seq and bulk RNA-seq dataset. **f,** The cell number profiled per cell line. **g/h,** Boxplots showing distributions of the number of UMI (g) /gene (h) detected per cell. For each boxplot, the center line represents the median, the box indicates the upper and lower quartiles, the whisker represents 1.5-fold of the interquartile range. **i,** Spearman correlation between the gene expression estimated from scRNA-seq (X-axis) and the bulk RNA-seq dataset of CCLE (or GEO) (Y-axis). **j,** Graphical representation of single-cell transcriptomics according to cancer type. **k,** UMAP plot of the number of UMI (log10) across all cell lines. **l,** UMAP plot of Library information of all cell lines. **m,** Graphical representation of cells of the same cell line in two independent experiments. **n,** Graphical representation of single-cell transcriptomics of colorectal cancer cell lines according to cancer subtype. Source data are provided in the Source Data file.

Supplementary Fig. 2

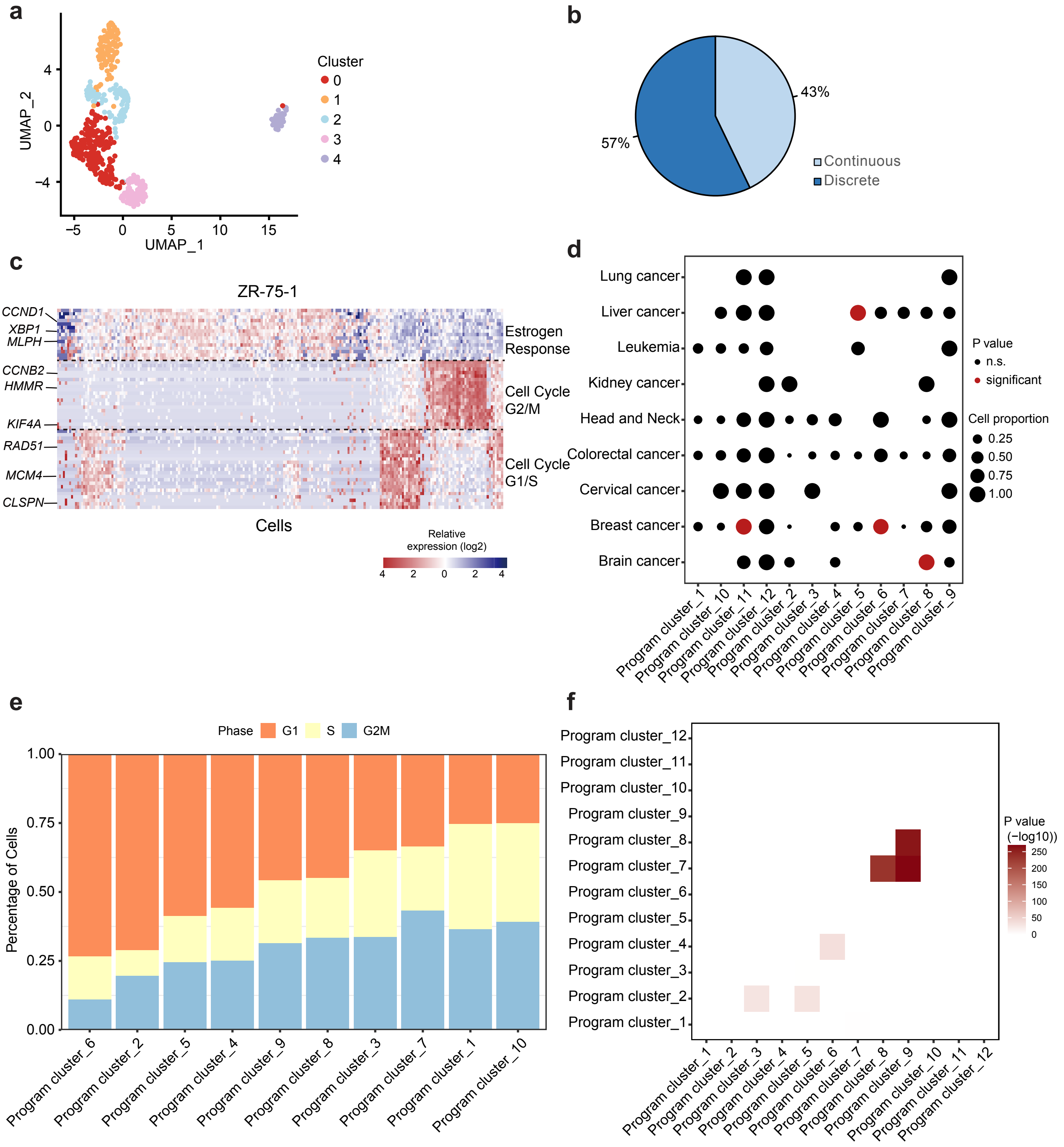

Supplementary Figure 2. Association of expression programs with cancer types and cell cycle.

a, Representative cell line SNB75 showing overall discrete pattern, different subclusters labeled by colors. **b,** Percentage of discrete and continuous cell lines. **c,** Heterogeneity programs identified using NMF in a representative cell line. The heatmap shows the relative gene expression of three NMF programs. NMF programs are annotated (right) and the selected marker genes are indicated (left). **d,** Dot plot shows the fraction of cell lines (n=42 cell lines) from each cancer type (rows) observed in each program cluster (columns). Circle size indicates the cell proportion. Red circles indicate those with hypergeometric test significance. **e,** Distribution of cells with high program cluster scores (top 5%) in different cell cycle phases. **f,** Co-occurrence of program clusters in the same cells (n=23,089 cells). A hypergeometric test was used to test the statistical significance. Source data are provided in the Source Data file.

Supplementary Fig. 3

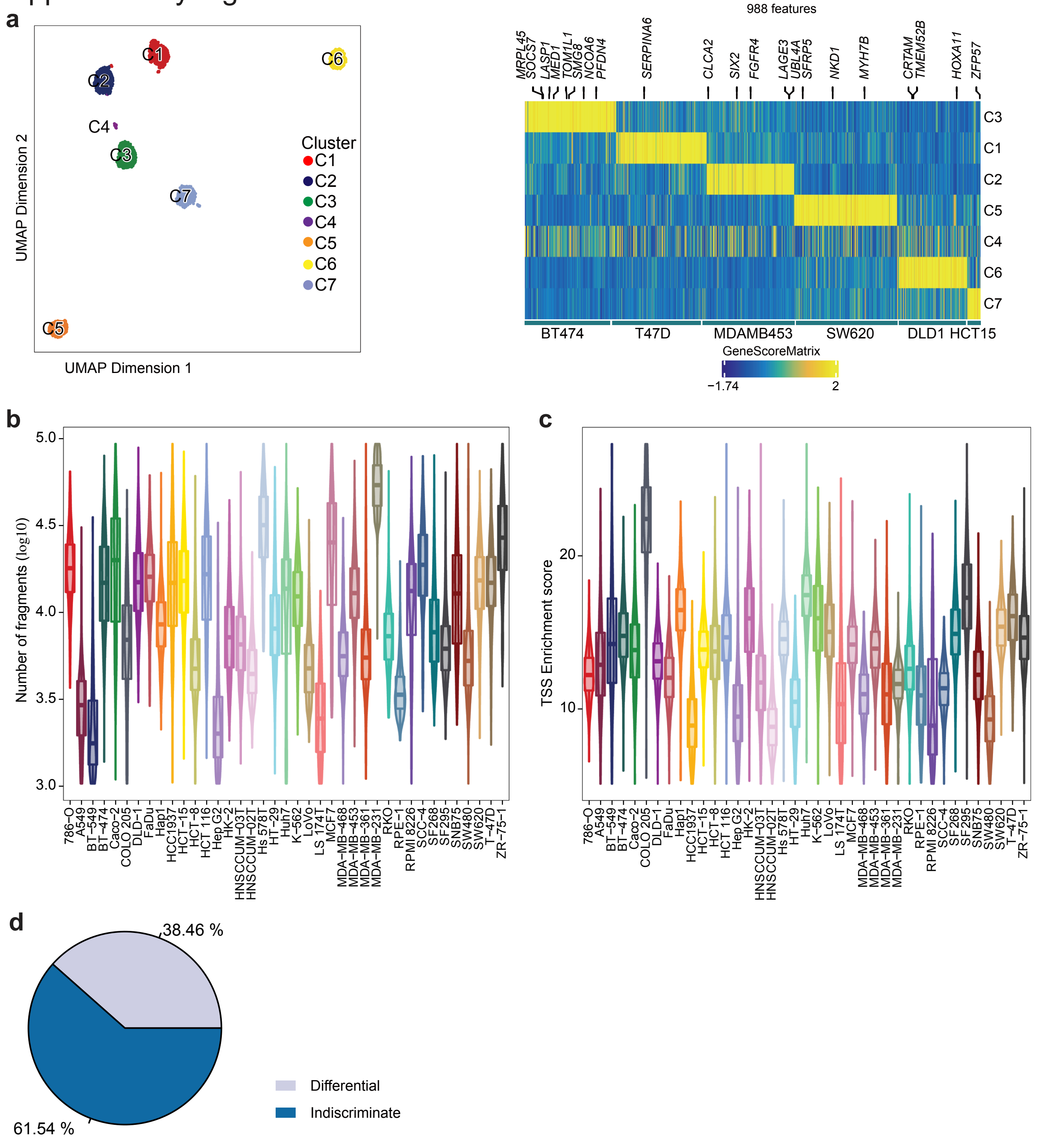

Supplementary Figure 3. Quality control of the scATAC-seq dataset

a, Clustering and cell lines assignment of a scATAC-seq pooling library. Left panel: UMAP plot of representative mix cells from the same scATAC-seq pooling library, cell clusters labeled by different colors. Right panel: Heatmap shows gene activity scores of marker genes across clusters calculated from scATAC-seq. Each column represents a unique marker gene. The color represents the normalized gene score of the marker genes in clusters. **b,** Boxplots show distributions of the number of fragments detected in each cell line. For each boxplot, the center line represents the median, the box indicates the upper and lower quartiles, the whisker represents 1.5-fold of the interquartile range. **c,** Boxplots show distributions of TSS enrichment score in each cell line. For each boxplot, the center line represents the median, the box indicates the upper and lower quartiles, the whisker represents 1.5-fold of the interquartile range. **d,** Percentage of differential and indiscriminate cell lines. Source data are provided in the Source Data file.

Supplementary Fig. 4a

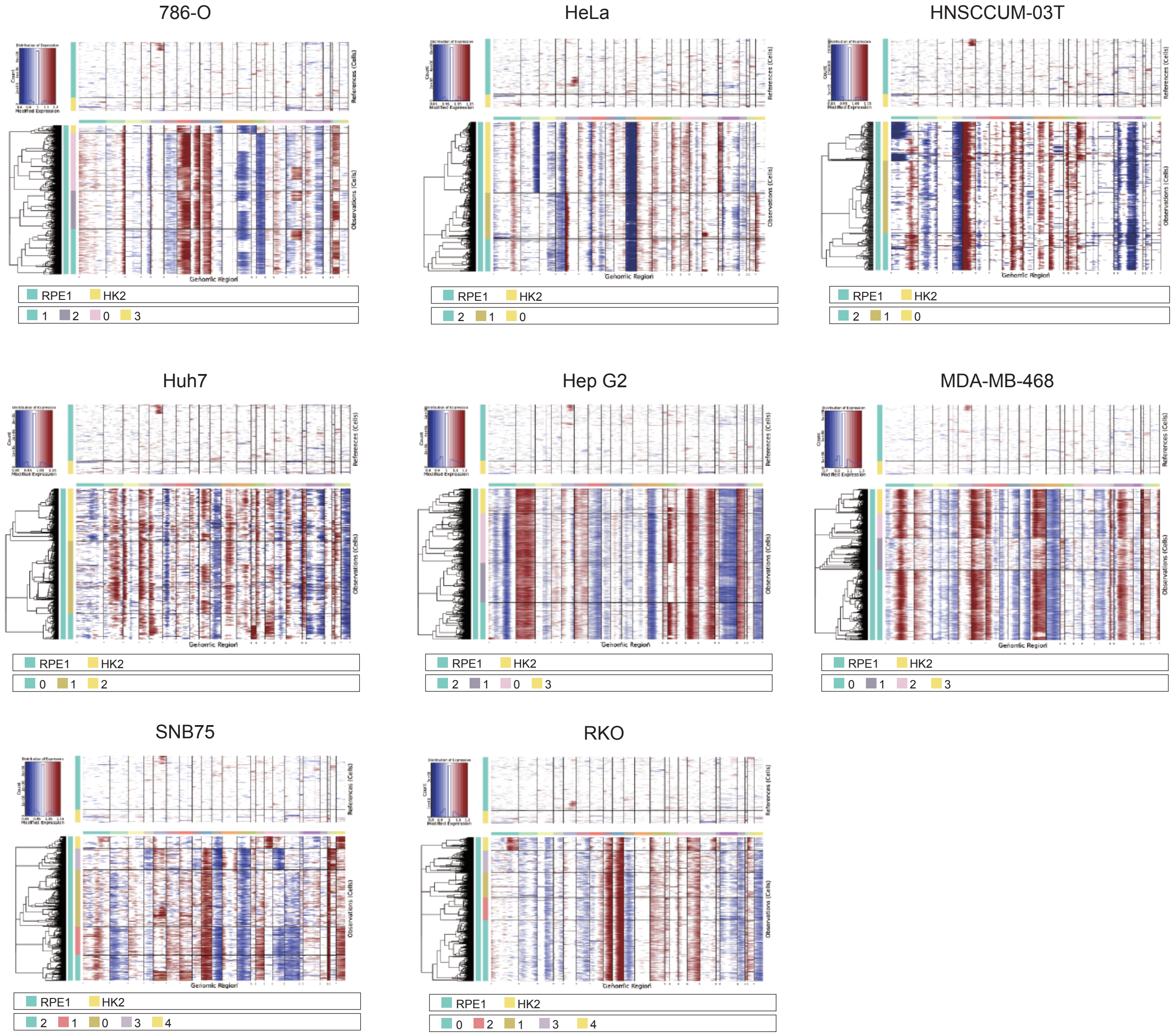

Supplementary Fig. 4b

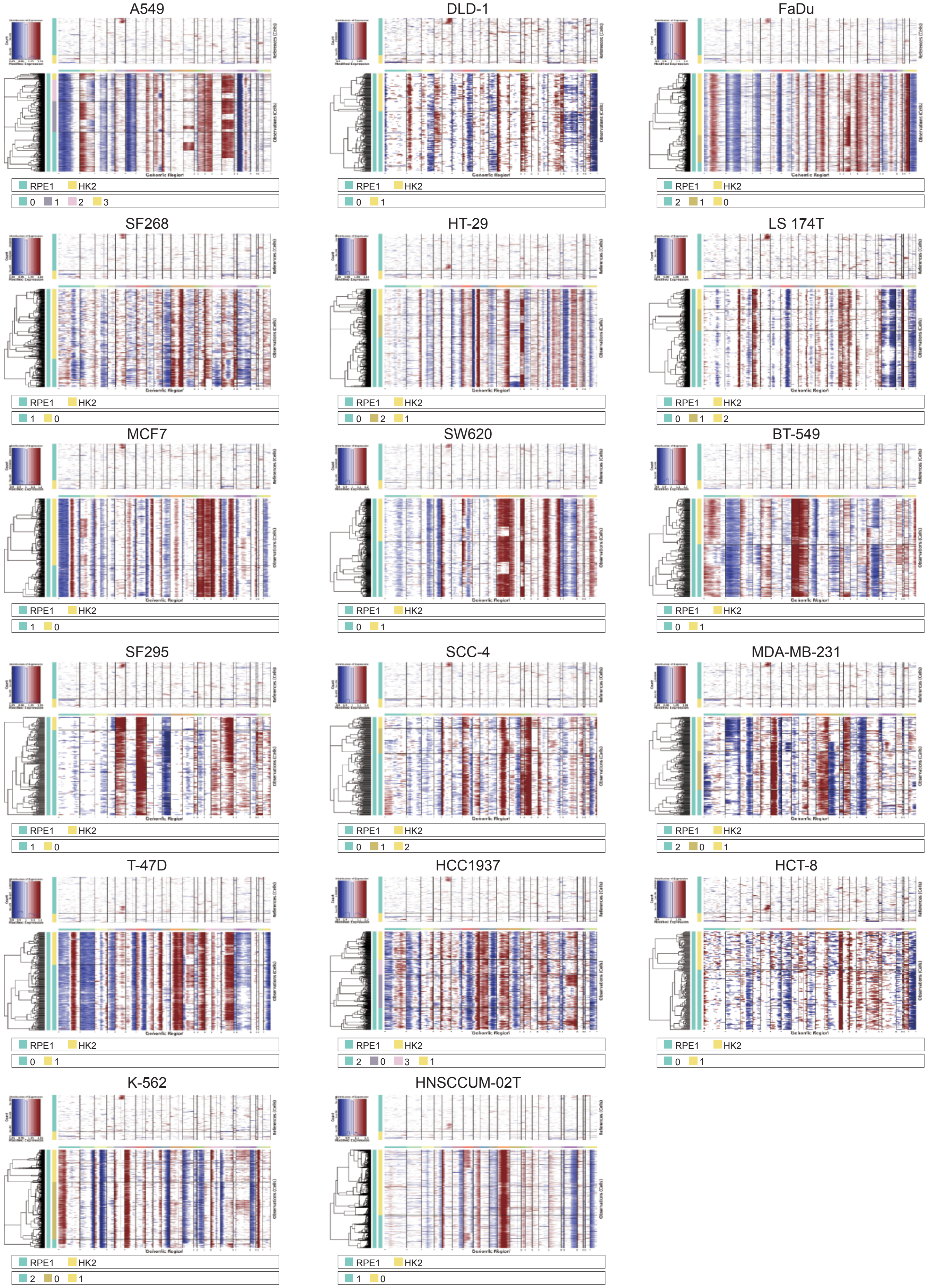

Supplementary Fig. 4c

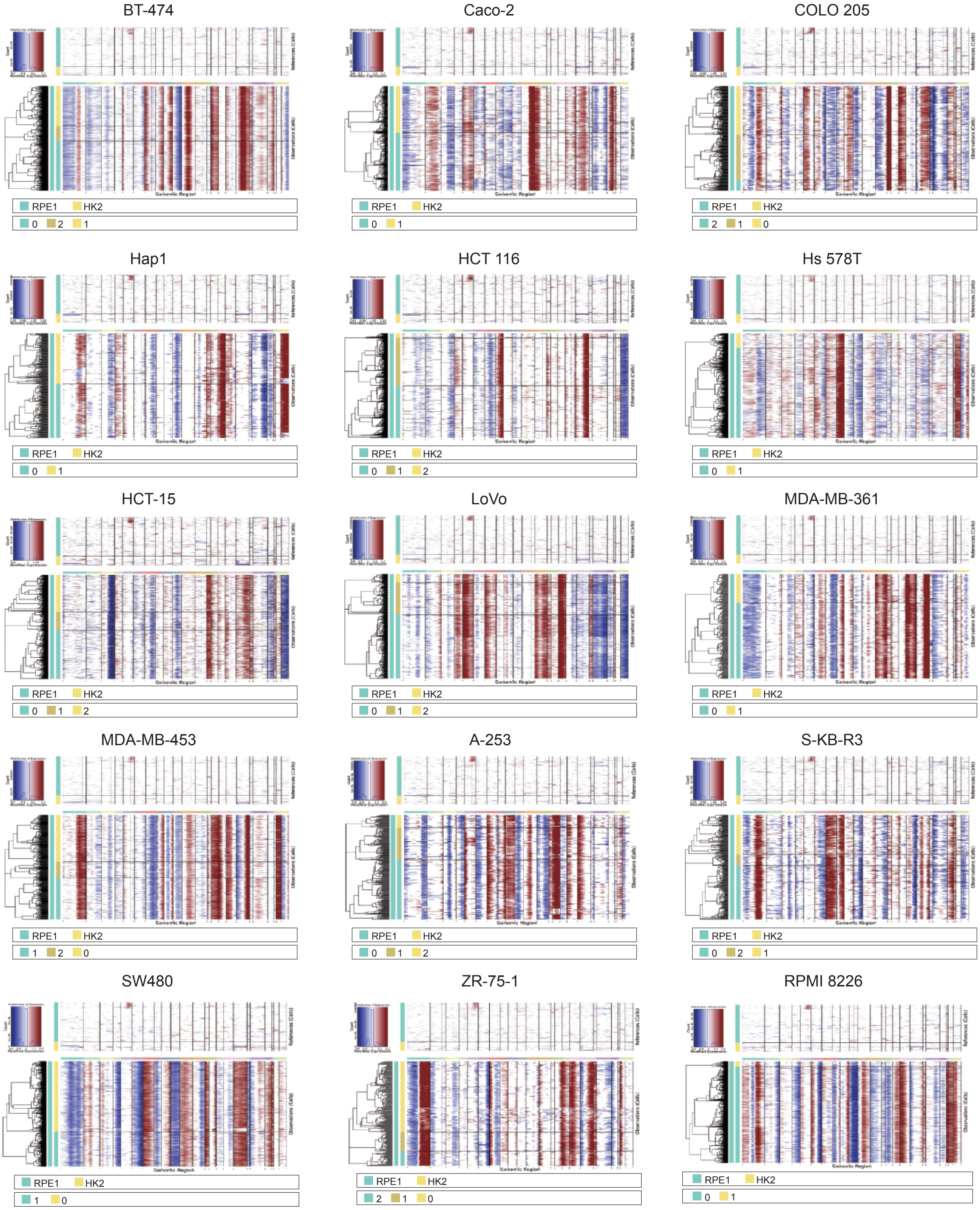

Supplementary Figure 4. CNVs inferred by scRNA-seq in all cell lines.

a, Cell lines that transcriptomic subcluster is linked to CNV sub-cluster. **b,** Cell lines showing that transcriptomic subcluster is not linked to CNV subcluster. **c,** Cell lines showing no CNV subcluster.

Supplementary Fig. 5

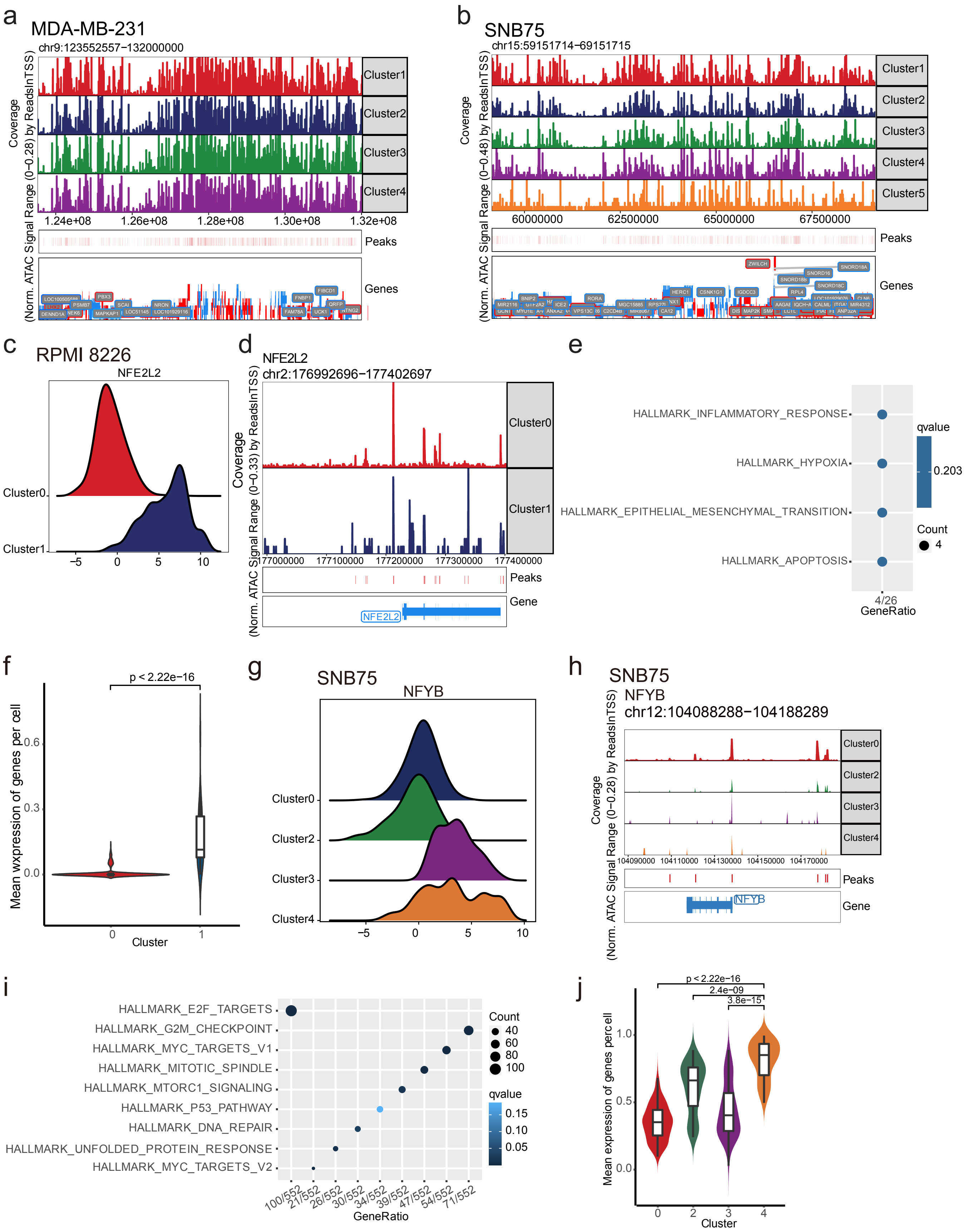

Supplementary Figure 5. Critical TFs for transcriptomic heterogeneity revealed by scATAC-seq data. **a-b,** The chromatin accessibility of CNV regions, which were selected according to scRNA-seq data, didn't show obvious heterogeneity across scATAC-seq subclusters in MDA-MB-231 (a) and SNB75 (b). **c,** Ridge plot showing activity of *NFE2L2* across subclusters in RPMI 8226. **d,** The chromatin accessibility of *NFE2L2* across subclusters in RPMI8226. **e,** Functional enrichment analysis of *NFE2L2* downstream targets with Hallmark gene set. A hypergeometric test was used to test the statistically significant differences. FDR-adjusted p-value<0.05. Q value for EMT is 0.22. **f,** The expression level of EMT-related genes among *NFE2L2* downstream targets in subclusters of RPMI 8226 (n=606 cells in cluster 0, n=32 cells in cluster 1). A two-sided Wilcoxon test was used to assess statistical significance. **g,** Ridge plot showing activity of *NFYB* across subclusters in SNB75. **h,** The chromatin accessibility of *NFYB* across subclusters in SNB75. **i,** Functional enrichment analysis of *NFYB* downstream targets with Hallmark gene set. A hypergeometric test was used to test the statistically significant differences. FDR-adjusted p-value<0.05. Q value for E2F_targets <0.01. **j,** The expression level of 'E2F targets' related genes among NFYB downstream targets in subclusters of SNB75 (n=614 cells in cluster 0, n=405 cells in cluster 2, n=47 in cluster 3, n=38 in cluster4). A two-sided Wilcoxon test was used to assess statistical significance. Source data are provided in the Source Data file.

Supplementary Fig. 6

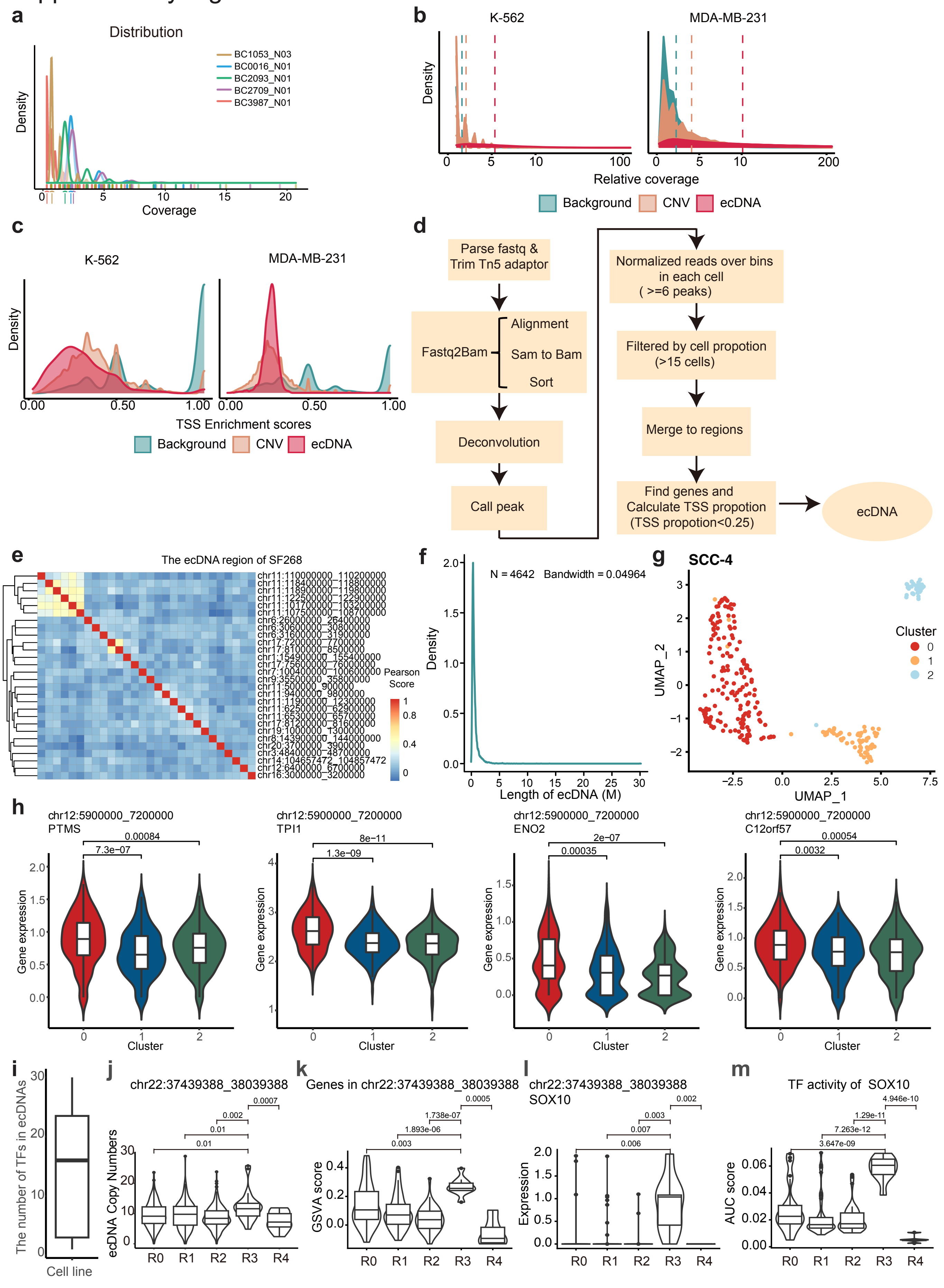

Supplementary Figure 6. The identification and length distribution of ecDNAs.

a, Representative result showing the read coverage across the genome of every single cell. The value of the horizontal coordinate represents the read coverage after normalization, the vertical coordinate represents the density, different colors represent different single cells, and the arrow under the coordinate axis indicates the location of the first peak of each cell. **b,** Distribution of the relative coverage regions of the different regions. The x-axis represents the relative coverage and the y-axis represents the density. The dotted line shows the mean value. The different colors stand for different regions: blue indicates the random region, yellow indicates the CNV region and red indicates the ecDNA region. **c,** Distribution of transcription start site enrichment scores for different regions. **d,** Schematic of analysis workflow for identifying the candidate region of ecDNA based on scATAC-seq data. **e,** Heatmap of ecDNA fragments correlation in SF268 cell line. **f,** The distribution of ecDNA length. **g,** UMAP plots of scRNA-seq subclusters of SCC4. **h,** The expression of genes located on ecDNA (chr12: 5900000_7200000) in different scRNA-seq subclusters of MDA-MB-231 (n=224 cells in cluster 0, n=878 cells in cluster 1, n=952 cells in cluster 2). A two-sided Wilcoxon test was used to assess statistical significance. For each boxplot, the center line represents the median, the box indicates the upper and lower quartiles, the whisker represents 1.5-fold of the interquartile range. **i,** The number distribution of TFs within ecDNAs across 39 cell lines. For each boxplot, the center line represents the median, the box indicates the upper and lower quartiles, the whisker represents 1.5-fold of the interquartile range. **j,** The copy number of ecDNA (chr22:37439388_38039388) across the five scRNA-seq sub-clusters of SNB75. A two-sided t test was used to test the statistical significance. For each boxplot, the center line represents the median, the box indicates the upper and lower quartiles, the whisker represents 1.5-fold of the interquartile range. **k,** The GSVA score of gene sets located on ecDNA (chr22:37439388_38039388) in different scRNA-seq sub-clusters of SNB75. A two-sided t test was used to test the statistical significance. For each boxplot, the center line represents the median, the box indicates the upper and lower quartiles, the whisker represents 1.5-fold of the interquartile range. **l,** The expression level of SOX10 in different clusters. A two-sided t test was used to test the statistical significance. For each boxplot, the center line represents the median, the box indicates the upper and lower quartiles, the whisker represents 1.5-fold of the interquartile range. **m,** The TF activity of SOX10 in different scRNA-seq subclusters of SNB75. The TF activity is represented by the area under the

recovery curve (AUC) score. A two-sided t test was used to test the statistical significance. For each boxplot, the center line represents the median, the box indicates the upper and lower quartiles, the whisker represents 1.5-fold of the interquartile range. Source data are provided in the Source Data file.

Supplementary Fig. 7

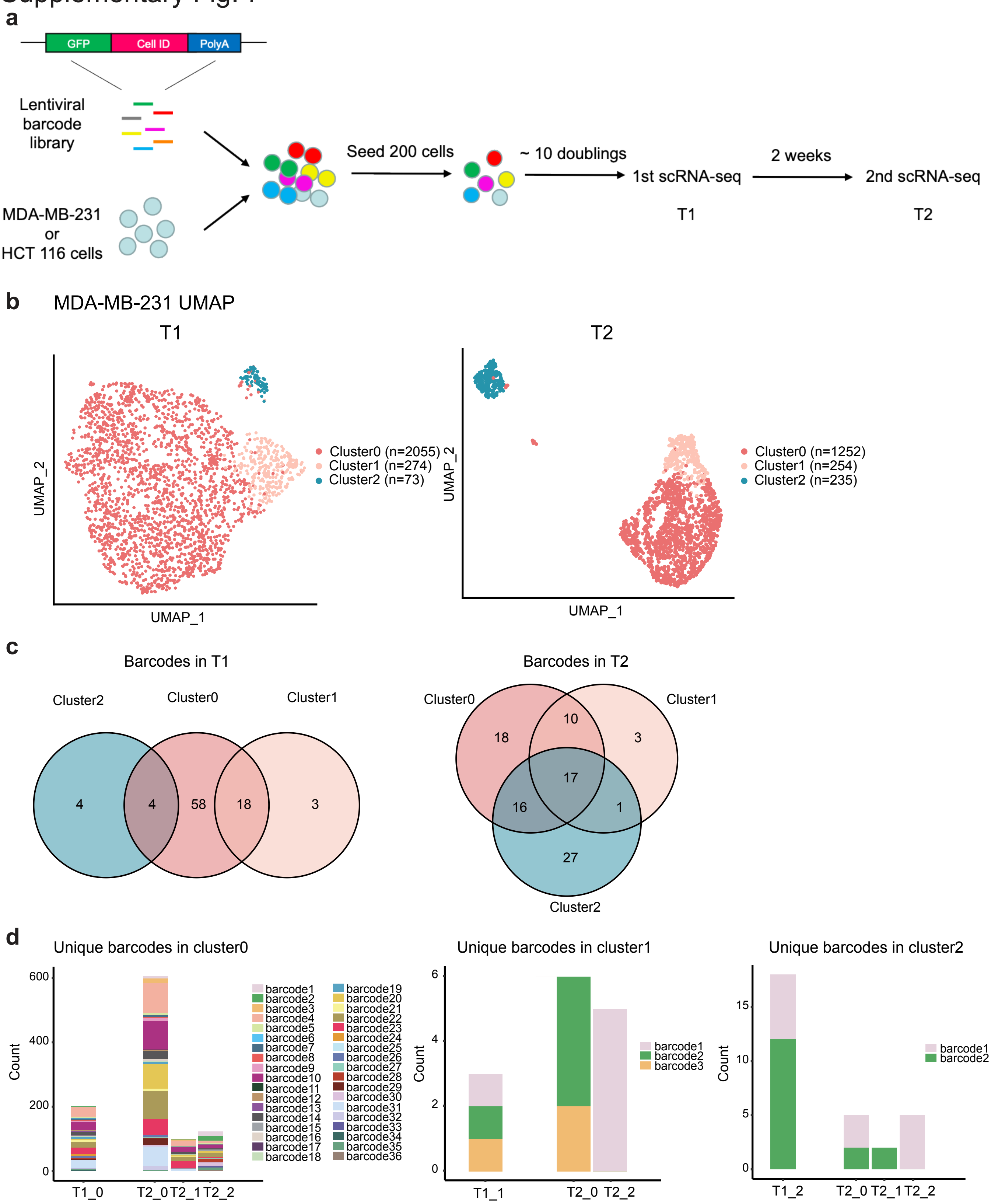

Supplementary Figure 7. The plasticity of transcriptomic heterogeneity within cell line.

a, The scheme of tracing experiment: cells were transfected with unique Cell-ID, then grown from 200 cells and analyzed in two different time points. **b,** UMAP plot of MDA-MB-231 at T1 and T2 (n=2402 cells at T1, n=1741 cells at T2). N represents cell numbers in different subgroups. **c,** Venn diagram of barcodes in different subclusters of MDA-MB-231 at T1 and T2. **d,** The distribution of unique barcodes in different subclusters at T1 was rearranged at T2 for MDA-MB-231. Timepoint combined with the original cluster number was indicated, e.g. T1_0 represented cluster0 from T1. Source data are provided in the Source Data file.

Supplementary Fig. 8

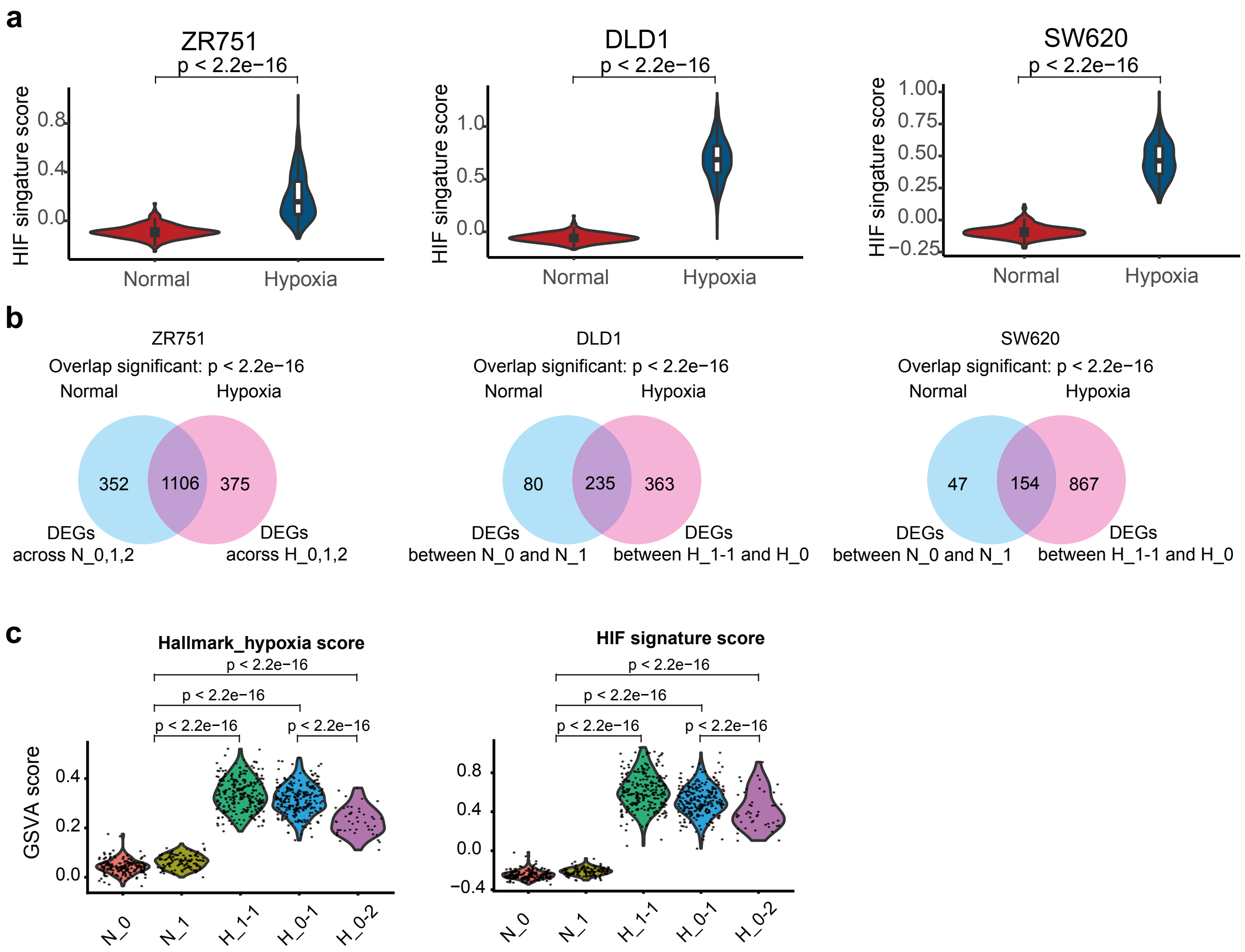

Supplementary Figure 8. The response of cells under the hypoxia.

a, Violin plot showing the GSVA score of HIF signature under the normal and hypoxia condition in ZR-75-1 (n=433 cell under hypoxia condition, n=208 under normal condition), DLD-1 (n=532 cell under hypoxia condition, n=289 under normal condition), and SW620 (n=250 cell under hypoxia condition, n=381 under normal condition). A two-sided Wilcoxon test was used to assess statistical significance. **b,** The overlap of DEGs identified between different subclusters under normoxia and DEGs identified between different subclusters under hypoxia in ZR751, DLD1, and SW620. A one-tailed hypergeometric test was used to test the statistical significance. **c,** The Module score distribution of Hypoxia and HIF signature in the sub-clusters under normal and hypoxia conditions (n=177 in cluster N_0, n=112 in cluster N_1, n=252 in cluster H_1-1, n=235 in cluster H_0-1, n=45 in cluster H_0-2). A two-sided Wilcoxon test was used to assess statistical significance. Source data are provided in the Source Data file.

Supplementary Table 1. Annotation of cell lines profiled.

Cell line name, lineage, number of cells profiled, pattern, and the relationship between transcriptional sub-cluster and CNV subclone.

**Name**	**Primary Disease**	**Cell No.**	**Pattern (Discrete/Continuous)***	**Relationship with CNV#**
HCT 116	Colon/colorectal cancer	543	C	C
HCT-15	Colon/colorectal cancer	947	C	C
HT-29	Colon/colorectal cancer	679	C	B
SW620	Colon/colorectal cancer	381	D	B
Caco-2	Colon/colorectal cancer	402/420	C	C
SW480	Colon/colorectal cancer	793	C	C
DLD-1	Colon/colorectal cancer	289	D	B
LS 174T	Colon/colorectal cancer	530	C	B
COLO 205	Colon/colorectal cancer	355	D	C
LoVo	Colon/colorectal cancer	424	C	C
HCT-8	Colon/colorectal cancer	207	D	B
RKO	Colon/colorectal cancer	560	D	A
MCF7	Breast cancer	409	C	B
MDA-MB-231	Breast cancer	299/506	D	B
MDA-MB-361	Breast cancer	228	C	C
BT-474	Breast cancer	752	D	C
MDA-MB-453	Breast cancer	396	D	C
BT-549	Breast cancer	327	D	B
T-47D	Breast cancer	262	C	B
MDA-MB-468	Breast cancer	885	D	A
Hs 578T	Breast cancer	496	D	C
HCC1937	Breast cancer	385	D	B
ZR-75-1	Breast cancer	221	D	C
SK-BR-3	Breast cancer	297	C	C
FaDu	Head and neck cancer	696	D	B
A-253	Head and neck cancer	274	C	C
SCC-4	Head and neck cancer	267/409	D	B
HNSCCUM-03T	Head and neck cancer	311	C	A
HNSCCUM-02T	Head and neck cancer	715	C	B
K-562	Leukemia	725	D	B
RPMI 8226	Leukemia	638	D	C
SF268	Brain cancer	414	D	B
SNB75	Brain cancer	542	D	A
SF295	Brain cancer	229	D	B
A549	Lung cancer	1451	C	B
Huh7	Liver cancer	462	D	A

Hep G2	Liver cancer	1115	D	A
786-O	Kidney cancer	896	D	A
Hap1	Leukemia	308	C	C
HeLa	Cervical cancer	787	D	A
RPE-1	Retina, non-tumor	693	C	
HK-2	Renal, non-tumor	164	C	

*: The pattern of transcriptional sub-clusters: we categorize cell lines into two patterns: Continuous (C) vs Discrete (D).

#: The relationship between transcriptional sub-cluster and CNV: We categorize these cell lines into three types: A. CNV sub-clones are linked to transcriptional sub-clusters (at least one sub-cluster); B. There are CNV sub-clones, but CNV sub-clones are not linked to transcriptional sub-clusters; C. There is no CNV sub-clone.

Supplementary Table 2. Annotation of molecular subtypes for mammary and colorectal cancer cell lines.

**Mammary cancer cell line**	**Subtype**	
MCF7	LA	
MDA-MB-231	TNB	
MDA-MB-361	LB	
BT-474	LB	
MDA-MB-453	H	
BT-549	TNB	
T-47D	LA	
MDA-MB-468	TNA	
Hs 578T	TNB	
HCC1937	TNA	
ZR-75-1	LA	
SK-BR-3	H	
We categorize breast cancer cell lines into luminal A (LA), luminal B (LB), HER2 positive (H), Triple negative A (TNA) and Triple negative B (TNB), according to literature available subtyping suggestions.		
**Colorectal cancer cell line**	**Subtype**	**Subtype defined in our analysis**
HCT 116	CMS4	CMS4
HCT-15	CMS1	CMS1
HT-29	CMS3	CMS3
SW620	CMS4	CMS4
Caco-2	CMS4	CMS4
SW480	CMS4	CMS4
DLD-1	CMS1	CMS3
LS 174T	CMS3	CMS3
COLO 205	CMS1	CMS1
LoVo	CMS1	CMS1
HCT-8	CMS4	CMS4
RKO	CMS4	CMS4
We categorize colorectal cancer cell lines into four consensus molecular subtypes: CMS1 (microsatellite instability immune), hypermutated, microsatellite unstable and strong immune activation; CMS2 (canonical), epithelial, marked WNT and MYC signaling activation; CMS3 (metabolic), epithelial and evident metabolic dysregulation; and CMS4 (mesenchymal), prominent transforming growth factor-β activation, stromal invasion and angiogenesis.		

Supplementary Table 3. Overlapped genes between our programs and published data (Kinker, Greenwald, et al. 2020).

Region	Meta-programs	Common program in our analysis	common genes
Region_1	Metabolism	NA	
Region_2	EMT II	EMT II	MYL9,SERPINE1,FN1,TAGLN,COL5A1,IGFBP3,INHBA,TPM1,VIM,FSTL1,IGFBP7
Region_3	IFN response	IFN Response	ISG20,IFIT3,ISG15,OASL,IFIT1,IFIT2,IFI44,PMAIP1,ZC3HAV1,HERC5,SAMD9,WARS,IGFBP6,IFI6,IFIH1,PARP14,HLA-B,B4GALT5,DDX58,DDX60,JUNB,KLF4,PLAUR,RSAD2,SDC4,APOL1,CDKN1A,NA
Region_4	Stress response	Stress Response	DDIT3,SLC3A2,PPP1R15A,GADD45A,TXNIP,TRIB3,ASNS,EPB41L4A-AS1,CARS,TSC22D1,DDIT4,HERPUD1,NA
Region_5	EMT I	EMT I	KRT7,PRSS23,IER3,S100A4,TNFRSF12A,CSRP1,FHL2,KRT18,S100A2,NA
Region_6	Estrogen response	NA	
Region_7	Mitotic spindle	NA	
Region_8	MT	NA	
Region_9	interphase	NA	
Region_10	MYC	NA	
Region_11	G1/S	G1/S	HIST1H4C,CLSPN,ATAD2,HELLS,RRM2,FAM111A,GINS2,CENPU,CDCA5,ASF1B,CHAF1A,TCF19,FEN1,PCNA,FBXO5,CDCA4,PKMYT1,FAM111B,USP1,CDC6,PSMC3IP,GMNN,ORC6,TYMS,CDK1,UBE2T,BRCA1,ESCO2,ATAD5,RAD51AP1,NA
Region_12	G2/M	G2/M	AURKA,CENPF,PLK1,TOP2A,UBE2C,ASPM,TPX2,CENPA,CKAP2,GTSE1,CCNB1,ARL6IP1,MKI67,CENPE,CKS2,HMMR,DEPDC1,NUSAP1,PRC1,CCNA2,KPNA2,CDCA8,HMGB2,NUF2,KNSTRN,CDCA3,CEP55,KIF20B,FAM83D,CDC20,DLGAP5,KIF2C,PRR11,ARHGAP11A,KIF23,AURKB,CDK1,KIF14,CCNB2,PSRC1,NEK2,CDCA2,BIRC5,TACC3,CKAP2L,HJURP,KIF4A,RACGAP1,ANLN,CDKN3,NA

Supplementary Table 4. Annotation of cell lines profiled in scATAC-seq.

Cell lines	Cell No.	Pattern
HCT 116	3075	Differential
HCT-15	1107	Indiscriminate
HT-29	1506	Indiscriminate
SW620	771	Indiscriminate
Caco-2	3303	Differential
SW480	1269	Indiscriminate
DLD-1	1070	Indiscriminate
LS 174T	203	Differential
COLO 205	1086	Indiscriminate
LoVo	955	Indiscriminate
HCT-8	1380	Indiscriminate
RKO	1364	Indiscriminate
MCF7	4391	Differential
MDA-MB-231	2054	Differential
MDA-MB-361	922	Indiscriminate
BT-474	1077	Indiscriminate
MDA-MB-453	1283	Indiscriminate
BT-549	627	Indiscriminate
T-47D	1113	Indiscriminate
MDA-MB-468	1211	Indiscriminate
Hs 578T	2664	Differential
HCC1937	1170	Indiscriminate
ZR-75-1	2489	Indiscriminate
FaDu	1374	Differential
SCC-4	1186	Indiscriminate
HNSCCUM-03T	1074	Differential
HNSCCUM-02T	1373	Differential
K-562	1482	Indiscriminate
RPMI 8226	1385	Differential
SF268	1138	Differential
SNB75	1104	Differential
SF295	1760	Indiscriminate
A549	800	Indiscriminate
Huh7	1254	Indiscriminate
Hep G2	643	Indiscriminate
786-O	1169	Differential
RPE-1	889	Differential
Hap1	926	Indiscriminate
HK-2	950	Differential

Supplementary Table 5. Annotation of cell lines profiled in hypoxia treatment.

**Cell lines**	**Cell No./scRNA-seq**
SW620	252
DLD-1	623
ZR-75-1	475
