# Supplementary material for: Single cell multi-omics reveal intra-cell-line heterogeneity across human cancer cell lines
Source: Nat Commun. 2023 Dec 9;14:8170. doi: 10.1038/s41467-023-43991-9 (PMC10710513; doi:10.1038/s41467-023-43991-9)
Supplement: Supplementary file 3 — Description of Additional Supplementary Files [file 41467_2023_43991_MOESM3_ESM.pdf]

Description of Additional Supplementary Files

Supplementary Data 1
Expression programs detected by NMF in each of the 42 cell lines analyzed.

Supplementary Data 2
Expression programs derived from NMF.

Supplementary Data 3
TF clusters revealed by scATAC-seq.

Supplementary Data 4
TFs sorted with the motif enriched in the peaks of heterogeneous accessibility.

Supplementary Data 5
The distribution of ecDNA in all cell lines.
