# Supplementary material for: Single cell multi-omics reveal intra-cell-line heterogeneity across human cancer cell lines
Source: Nat Commun. 2023 Dec 9;14:8170. doi: 10.1038/s41467-023-43991-9 (PMC10710513; doi:10.1038/s41467-023-43991-9)
Supplement: Supplementary file 9 — Reporting Summary [file 41467_2023_43991_MOESM9_ESM.pdf]

nature portfolio

Corresponding author(s): Wei Chen

Last updated by author(s): Nov 19, 2023

Reporting Summary

Nature Portfolio wishes to improve the reproducibility of the work that we publish. This form provides structure for consistency and transparency in reporting. For further information on Nature Portfolio policies, see our Editorial Policies and the Editorial Policy Checklist.

Statistics

For all statistical analyses, confirm that the following items are present in the figure legend, table legend, main text, or Methods section.

n/a	Confirmed	
☐	☒	The exact sample size (*n*) for each experimental group/condition, given as a discrete number and unit of measurement
☐	☒	A statement on whether measurements were taken from distinct samples or whether the same sample was measured repeatedly
☐	☒	The statistical test(s) used AND whether they are one- or two-sided<br>*Only common tests should be described solely by name; describe more complex techniques in the Methods section.*
☒	☐	A description of all covariates tested
☐	☒	A description of any assumptions or corrections, such as tests of normality and adjustment for multiple comparisons
☐	☒	A full description of the statistical parameters including central tendency (e.g. means) or other basic estimates (e.g. regression coefficient) AND variation (e.g. standard deviation) or associated estimates of uncertainty (e.g. confidence intervals)
☐	☒	For null hypothesis testing, the test statistic (e.g. F, t, r) with confidence intervals, effect sizes, degrees of freedom and P value noted<br>*Give P values as exact values whenever suitable.*
☒	☐	For Bayesian analysis, information on the choice of priors and Markov chain Monte Carlo settings
☒	☐	For hierarchical and complex designs, identification of the appropriate level for tests and full reporting of outcomes
☐	☒	Estimates of effect sizes (e.g. Cohen's *d*, Pearson's *r*), indicating how they were calculated

Our web collection on statistics for biologists contains articles on many of the points above.

Software and code

Policy information about availability of computer code

Data collection	Single-cell RNA and ATAC sequencing data collection was performed on BGISEQ500 sequencer or DIPSEQ T1 sequencer.
Data analysis	Data analysis was performed using inferCNV (v1.8.1), Seurat (v4.1.0), PISA (v0.4), STAR (v.2.7.9), NMF, BWA (v.2.2), MACS2 (v2.2.7.1), ArchR (v.1.0.2), ChromVAR (v.1.16.0), SCENIC, pySCENIC (v 0.12.0), cBioPortal, ClusterProfiler (v4.2.2), GSVA (v1.44.2), and Limma (v.3.50.0). Custom code used is available from GitHub at https://github.com/liushang17/CCLA. A full description of data analysis is found in 'Methods'.

For manuscripts utilizing custom algorithms or software that are central to the research but not yet described in published literature, software must be made available to editors and reviewers. We strongly encourage code deposition in a community repository (e.g. GitHub). See the Nature Portfolio guidelines for submitting code & software for further information.

Data

Policy information about availability of data

All manuscripts must include a data availability statement. This statement should provide the following information, where applicable:

- Accession codes, unique identifiers, or web links for publicly available datasets
- A description of any restrictions on data availability
- For clinical datasets or third party data, please ensure that the statement adheres to our policy

The bulk cancer cell line RNA-seq data used in this study are publicly available through depmap portal at https://depmap.org/portal and GEO (SKBR3: under accession code GSE7562 at https://www.ncbi.nlm.nih.gov/geo/query/acc.cgi?acc=GSE7562; LS174T: under accession code GSE18560 at https://

www.ncbi.nlm.nih.gov/geo/query/acc.cgi?acc=GSE18560). The scRNA-seq and scATAC-seq data generated in this study have been deposited in China National GeneBank DataBase (CNGBdb) Sequence Archive (CNSA) with accession number CNP0004330 (https://db.cngb.org/search/project/CNP0004330/) and also in the Genome Sequence Archive (GSA) database under accession number PRJCA021248 (https://ngdc.cncb.ac.cn/bioproject/browse/PRJCA021248). Processed scRNA-seq and scATAC-seq data have been available in CNSA with accession number CNP0003658 (https://db.cngb.org/search/project/CNP0003658/) and also in GSA under accession number PRJCA020910 (https://ngdc.cncb.ac.cn/bioproject/browse/PRJCA020910). We also supply the CCLA website (https://db.cngb.org/cdcp/scatlashcl/), an open and interactive database for exploration. The remaining data are available within the Article, Supplementary Information or Source Data file.

Human research participants

Policy information about studies involving human research participants and Sex and Gender in Research.

Reporting on sex and gender	N/A
Population characteristics	N/A
Recruitment	N/A
Ethics oversight	N/A

Note that full information on the approval of the study protocol must also be provided in the manuscript.

Field-specific reporting

Please select the one below that is the best fit for your research. If you are not sure, read the appropriate sections before making your selection.

☒ Life sciences ☐ Behavioural & social sciences ☐ Ecological, evolutionary & environmental sciences

For a reference copy of the document with all sections, see nature.com/documents/nr-reporting-summary-flat.pdf

Life sciences study design

All studies must disclose on these points even when the disclosure is negative.

Sample size	No statistical methods were used to predetermine sample size. The study included 42 human cell lines. Sample size was determined by the availability of cell lines.
Data exclusions	In the scRNA-seq data, we excluded cells with below 1000 UMI and/or 500 genes. In the scATAC-seq data, cells with a low number of TSS proportion (<4) and a low number of unique fragments (<1000) were filtered out. Moreover, cells identified as doublets, low quality cells, or with inconsistent assignment between the bulk expression and CNA-based methods were further excluded.
Replication	All the computational results were replicable by running our algorithm twice. To validate the the reproducibility of our scRNA-seq experiment, we analyzed three cell lines in two independent experiments (as shown in supplementary Figure 1m). The results were successfully replicated.
Randomization	We did not apply randomization of the samples because no intervention was conducted in our study.
Blinding	Blinding was not used in this study, as sample information was necessary in order to perform analysis.

Reporting for specific materials, systems and methods

We require information from authors about some types of materials, experimental systems and methods used in many studies. Here, indicate whether each material, system or method listed is relevant to your study. If you are not sure if a list item applies to your research, read the appropriate section before selecting a response.

Materials & experimental systems

n/a	Involved in the study	
☒	☐	Antibodies
☐	☒	Eukaryotic cell lines
☒	☐	Palaeontology and archaeology
☒	☐	Animals and other organisms
☒	☐	Clinical data
☒	☐	Dual use research of concern

Methods

n/a	Involved in the study	
☒	☐	ChIP-seq
☒	☐	Flow cytometry
☒	☐	MRI-based neuroimaging

Eukaryotic cell lines

Policy information about cell lines and Sex and Gender in Research

Cell line source(s)	The 786-O (CRL-1932), A253 (HTB-41), A549 (CCL-185), BT-474 (HTB-20), BT-549 (HTB-122), Caco-2 (HTB-37), COLO 205 (CCL-222), DLD-1 (CCL-221), FaDu (HTB-43), HCC1937 (CRL-2336), HCT 116 (CCL-247), HCT-15 (CCL-225), HCT-8 (CCL-244), HeLa (CCL-2), Hep G2 (HB-8065), HK-2 (CRL-2190), Hs 578T (HTB-126), HT-29 (HTB-29), K-562 (CCL-243), LoVo (CCL-229), LS 174T (CL-188), MCF7 (HTB-22), MDA-MB-231 (HTB-26), MDA-MB361 (HTB-27), MDA-MB-453 (HTB-131), MDA-MB-468 (HTB-132), RKO (CRL-2577), RPE-1 (CRL-4000), RPMI 8226 (CCL-155), SCC4 (CRL-1624), SK-BR-3 (HTB-30), SW480 (CCL-228), SW620 (CCL-227), T-47D (HTB-133) and ZR-75-1 (CRL-1500) cells were obtained from the ATCC. The HAP1 (C631) cells are from Horizon Discovery and Huh7 (SCSP-526) cells are from The Cell Bank of the Chinese Academy of Sciences. The SF268, and SNB75 cells are from NCI, and the SF295 (C0005005) cells are from AddexBio. The HUNSCCUM-02T and HUNSCCUM-03T cells are kindly gifted by Walter Birchmeier's lab at Max-Delbrueck-Center for molecular Medicine, Berlin.
Authentication	We pick eight cell lines for authentication. Cell line identity was confirmed by the Multiplex human cell line Authentication Test (MCA, Mutiplexion) or Cell Line Authentication (GENEWIZ, Suzhou).
Mycoplasma contamination	All cell lines tested negative for mycoplasma.
Commonly misidentified lines (See ICLAC register)	No commonly misidentified lines were used in this study.
